# Metabolomic Profiling Reveals That Exercise Lowers Biomarkers of Cardiac Dysfunction in Rats with Type 2 Diabetes

**DOI:** 10.3390/antiox13101167

**Published:** 2024-09-26

**Authors:** Tutu Wang, Miaomiao Ning, Yurou Mo, Xinyu Tian, Yu Fu, Ismail Laher, Shunchang Li

**Affiliations:** 1 Institute of Sports Medicine and Health, Chengdu Sport University, Chengdu 610041, China; wtt0306@126.com (T.W.); 15662088661@163.com (M.N.); 18508189746@163.com (Y.M.); t1138790522@163.com (X.T.); flytogether0909@163.com (Y.F.); 2 Department of Anesthesiology, Pharmacology and Therapeutics, Faculty of Medicine, University of British Columbia, Vancouver, BC V6T 1Z3, Canada; ismail.laher@ubc.ca

**Keywords:** type 2 diabetes, cardiac function, aerobic exercise, metabolomics

## Abstract

The increasing prevalence of type 2 diabetes mellitus (T2DM) leads to significant global health challenges, including cardiac structural and functional deficits, which in severe cases can progress to heart failure that can further strain healthcare resources. Aerobic exercise can ameliorate cardiac dysfunction in individuals with diabetes, although a comprehensive understanding of its underlying mechanisms remains elusive. This study utilizes untargeted metabolomics to reveal aerobic-exercise-activated metabolic biomarkers in the cardiac tissues of Sprague Dawley rats with T2DM. Metabolomics analysis revealed that diabetes altered 1029 myocardial metabolites, while aerobic exercise reversed 208 of these metabolites, of which 112 were upregulated and 96 downregulated. Pathway topology analysis suggested that these metabolites predominantly contributed to purine metabolism and arginine biosynthesis. Furthermore, receiver operating characteristic curve analysis identified 10 potential biomarkers, including xanthine, hypoxanthine, inosine, dGMP, l-glutamic acid, l-arginine, l-tryptophan, (R)-3-hydroxybutyric acid, riboflavin, and glucolepidiin. Finally, data from Pearson correlation analysis indicated that some metabolic biomarkers strongly correlated with cardiac function. Our data suggest that certain metabolic biomarkers play an important role in ameliorating diabetes-related cardiac dysfunction by aerobic exercise.

## 1. Introduction

The global incidence of type 2 diabetes mellitus (T2DM) is increasing at an alarming rate, and is estimated to affect over 700 million patients worldwide by 2045 [1]. Diabetes is a metabolic disorder characterized by elevated blood glucose levels due to either insulin deficiency or insulin resistance [2]. Persistent hyperglycemia leads to insulin resistance, mitochondrial dysfunction, oxidative stress, myocardial fibrosis, β-adrenergic receptor downregulation, and other detrimental consequences that can contribute to cardiac dysfunction, which can lead to heart failure [3,4,5]. The pathophysiology of diabetes-related cardiac dysfunction is not well understood, and current treatment options are primarily centered on traditional strategies including extended caloric restriction, blood glucose and lipid management, and insulin resistance control [6].

Exercise is a favored approach for managing the health of individuals with T2DM, given its potential to ameliorate insulin resistance and reduce blood glucose levels [7]. Moreover, exercise has long been known to provide cardiovascular benefits while also being an important approach to manage heart disease [8]. Exercise enhances cardiorespiratory fitness, boosts ejection fraction and cardiac output, reduces the mortality rate of T2DM and the incidence of diabetic cardiac diseases [9]. Prolonged aerobic exercise in a rodent model of T2DM ameliorated cardiac structural and functional changes by augmenting myocardial glucose uptake, mitigating mitochondrial dysfunction, and diminishing myocardial fibrosis [10]. In addition, exercise training can increase the expression of β1-adrenergic receptors in the myocardium, thereby enhancing the myocardial responsiveness to catecholamine [11].

Metabolomics is a sensitive method of identifying endogenous small molecules in cells, bodily fluids, and tissues. For example, metabolites identified through metabolomics could be used as disease biomarkers useful for predicting and treating T2DM [12]. Using metabolomics techniques, researchers observed disrupted energy metabolism in T2DM patients. However, after engaging in various forms of exercise, such as running, tai chi, and brisk walking, improvements were noted in metabolic processes, including branched-chain amino and arachidonic acid metabolism [13,14]. Notably, metabolomic screening employing ultra-high-performance liquid chromatography coupled with quadruple time-of-flight mass spectrometry (UPLC-Q-TOF-MS) allows for improved assessment of metabolic processes, for example, by providing additional insights into the physiological and pathological pathways of T2DM [15,16].

We used untargeted metabolomics to assess the metabolic features of cardiac tissue from rats with T2DM subjected to aerobic exercise. Our study tested the hypothesis that aerobic exercise improves cardiac structure and function in a rat model of T2DM by altering metabolites and cardiac biomarkers. The findings of our study could be used to identify novel therapeutic targets to improve diabetic cardiac function based on the metabolic effects of aerobic exercise.

## 2. Materials and Methods

### 2.1. Experimental Animals

Eight-week-old male Sprague Dawley (SD) rats were purchased from Chengdu Dashuo Experimental Animal Co., Ltd. (Chengdu, China). The rats had body weights ranging from 200 to 210 g and were maintained under the following conditions: an ambient temperature of 21–23 °C, relative humidity of 40–60%, and a 12 h light/dark cycle. All rats had unrestricted access to both food and water. The experimental protocol received approval from the Academic Committee of Chengdu Sport University (Approval No: 2022-3).

A total of 40 rats were divided into two groups: the control group (C, *n* = 10) and the prediabetic model group (PD, *n* = 30). Rats with prediabetes were randomly divided into diabetic (D) and diabetic exercise (DE) subgroups.

### 2.2. STZ-Induced Rat Model of T2DM

Thirty rats were fed a high-sugar and high-fat diet for seven weeks, followed by intraperitoneal injection of streptozotocin (STZ, 30 mg/kg) that was dissolved in sodium citrate buffer (0.1 mol/L, pH = 4.4) [17,18]. Rats in group C were administered an intraperitoneal injection of sodium citrate buffer (0.25 mL/kg). Rats were considered to be diabetic when blood glucose levels reached ≥16.7 mmol/L 72 h after being injected with STZ injection [19]. Approximately 70% of the rats were successfully induced with T2DM.

Normal rat feed consisted of 5% fat, 50% carbohydrates, and 23% protein. High-fat feed included 67% regular feed, 10% lard, 20% sucrose, 2% cholesterol, and 1% sodium cholate [20]. Both regular feed and high-fat feed were provided by Chengdu Dashuo Biotechnology Co., Ltd. (Chengdu, China).

### 2.3. Exercise Protocol

Aerobic exercise was performed on a rat treadmill using the Bedford exercise model [21], with formal exercise starting after a 3-day adaptation period. Rats in the DE group ran on the treadmill, beginning at a speed of 8 m/min that was gradually escalated to 15 m/min, and maintained at this speed for 1 h daily, 5 days a week, for 8 weeks.

### 2.4. Assessment of Blood Glucose, Insulin and Insulin Resistance Index

Plasma glucose levels were determined using a blood glucose meter (ACON Biotech, Hangzhou, China). Insulin levels were measured using a rat insulin ELISA kit (ImmunoWay Biotechnology Company, San Jose, CA, USA) and an enzyme immunoassay reader set at 450 nm (SpectraMax M5 from Thermo Fish Scientific, Waltham, MA, USA). The insulin resistance index was computed using the following formula: HOMA-IR = Fasting Blood Glucose (FBG) (mmol/L) × Fasting Insulin (FINS) (mU/L)/22.5 [22].

### 2.5. Histological Staining

Myocardial tissue was obtained from 4% paraformaldehyde (Biosharp, Hefei, China) fixed tissues that were embedded in paraffin and stained with hematoxylin–eosin (HE). Following sectioning, the tissue slices underwent deparaffinization, hematoxylin staining, differentiation, eosin counterstaining, dehydration, xylene-based air-drying, and mounting with neutral resin. Rat myocardial tissue histology was examined using an optical microscope (Olympus Corporation, Tokyo, Japan).

### 2.6. Echocardiography

After anesthetizing the rats, cardiac structure and function were assessed using small animal echocardiography (Philips, Shanghai, China). The following cardiac parameters were assessed: left ventricular ejection fraction (EF), early-to-late diastolic filling velocity ratio (E/A), left ventricular end-diastolic internal diameter (LVIDd), left ventricular end-systolic internal diameter (LVIDs), left ventricular posterior wall end-diastolic thickness (LVPWd), and left ventricular posterior wall end-systolic thickness (LVPWs).

### 2.7. UPLC-Q-TOF/MS Analysis

Myocardial tissues from the rats under study end point were immediately frozen and stored at −80 °C. Upon thawing, 50 mg of myocardial tissue was weighed and 1000 μL of extraction solution containing an internal standard (methanol:acetonitrile:water = 2:2:1, internal standard concentration 20 mg/L) was added, followed by vortexing for 30 s. Steel beads were then added, and the sample was subjected to ultrasonic grinding at 45 Hz for 10 min in an ice-water bath. The tissue was centrifuged at 12,000 rpm for 15 min at 4 °C. Next, 500 μL of the supernatant was transferred to an EP tube and dried in a vacuum concentrator. The dried metabolites were reconstituted in 160 μL of extraction solution (acetonitrile:water = 1:1), vortexed for 30 s, and sonicated in an ice-water bath for 10 min. The sample was centrifuged again at 12,000 rpm for 15 min at 4 °C. Finally, 120 μL of the supernatant was transferred to a 2 mL vial, and 10 μL from each sample was prepared for analysis.

Myocardial metabolomics were analyzed via a liquid chromatography–mass spectrometry (LC-MS) system that featured a Waters UPLC Acquity I-Class PLUS high-performance liquid chromatography instrument (Waters Corporation, Milford, MA, USA) linked to a Waters UPLC Xevo G2-XS QTOF high-resolution mass spectrometer (Waters Corporation, Milford, MA, USA). Additionally, it was furnished with a Waters Acquity UPLC HSS T3 chromatographic column (1.8 μm, 2.1 mm × 100 mm) (Waters Corporation, Milford, MA, USA). The mobile phase solvents for the liquid chromatography consisted of the following: 0.1% formic acid aqueous solution (A) and 0.1% formic acid acetonitrile solution (B). The mobile phase conditions were as follows: (a) 0–0.25 min, 98% (A) and 2% (B); (b) 0.25–10 min, a gradient from 98% (A) and 2% (B) to 2% (A) and 98% (B); (c) 10–13 min, 2% (A) and 98% (B); (d) 13–13.1 min, a gradient from 2% (A) and 98% (B) to 98% (A) and 2% (B); (e) 13.1–15 min, 98% (A) and 2% (B). The sample injection volume was 1 μL, and the flow rate was maintained at 400 μL/min.

High-resolution mass spectrometry data acquisition was controlled using MassLynx V4.2 software (Waters Corporation, Milford, MA, USA). The mass-to-charge ratio (*m*/*z*) range for data collection was configured from 50 to 1200. The electrospray ionization (ESI) source was characterized by the following parameters: capillary voltage set at 2500 V or −2000 V, cone voltage at 30 V, ion source temperature at 100 °C, desolvation gas temperature at 500 °C, nebulizer gas flow rate of 50 L/h, and desolvation gas flow rate of 800 L/h.

### 2.8. Data Pre-Processing

Raw data obtained from MassLynx V4.2 were imported into the Progenesis QI V3.0 software for tasks such as peak detection and alignment. Identification was conducted using the Progenesis QI software against the METLIN database, HMDB database, and KEGG database, with theoretical fragment identification performed simultaneously. A mass error tolerance of 100 ppm was applied for precursor ions, and 50 ppm for fragment ions. Subsequent analysis ensued following the normalization of raw peak area data against the total peak area.

### 2.9. Statistical Analysis

Data were processed using MetaboAnalyst 5.0 software (https://www.metaboanalyst.ca, accessed on 15 December 2023). This involved eliminating missing values and logarithmic transformation of the metabolite data. Subsequently, differential metabolites underwent topological analysis and enrichment analysis via the ‘Pathway Analyst’ and ‘Enrichment Analyst’ modules. Receiver operating characteristic (ROC) curves were generated using OmicStudio tools (https://www.omicstudio.cn/tool, accessed on 18 December 2023). Prior to performing Pearson correlation analysis and one-way analysis of variance (ANOVA), we assessed the normality and homogeneity of variances for the data. Pearson correlation analysis was then conducted to evaluate the relationships between metabolite concentrations and echocardiographic indices, while group differences were analyzed using ANOVA, utilizing SPSS 26.0 or GraphPad prism 8. Data are expressed as means ± standard deviation (SD), with statistical significance defined as *p* < 0.05.

## 3. Results

### 3.1. Aerobic Exercise and Myocardial Weight, Insulin Resistance and Myocardial Structure in Diabetic Rats

**Figure 1:**
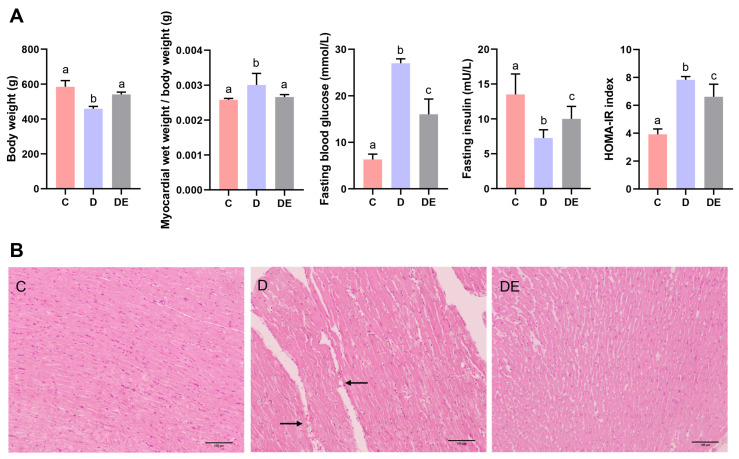
Changes in body weight, myocardial wet weight-to-body weight ratio, fasting blood glucose, fasting insulin, HOMA-IR index, and myocardial structure in T2DM rats after 8 weeks of aerobic exercise intervention. (**A**) Rats from group D had increased myocardial wet weight/body weight ratios compared to rats from group C (*p* < 0.01), whereas exercise reduced this ratio in diabetic rats (*p* < 0.01). Blood glucose and insulin resistance levels were higher in diabetic rats (*p* < 0.01), which were reduced by aerobic exercise (*p* < 0.05). (**B**) Representative histological HE staining images of myocardial longitudinal sections from different rat groups (×100), with a scale bar of 100 μm. The area indicated by the arrow shows inflammatory infiltration and vacuolar degeneration in rat cardiomyocytes. Different letters indicate significant differences between groups (*p* < 0.05), groups with the same letter are not significantly different (*p* > 0.05). HOMA-IR = homeostatic model assessment for insulin resistance; HE = hematoxylin–eosin. Numerical values are presented as means ± standard deviation. C, control group; D, diabetic group; DE, diabetic exercise group.

The body weights of rats in group D were 21.5% lower than those in group C (*p* < 0.01). However, after 8 weeks of aerobic exercise, rats in group DE had body weights that were 17.9% higher than those in group D, and had body weights similar to those in group C (*p* > 0.05). Additionally, myocardial wet weight/body weight ratios increased by 16.6% in diabetic rats, indicating myocardial hypertrophy (*p* < 0.01) (Figure 1A). Fasting blood glucose levels and the HOMA-IR index were higher in rats from group D than rats in group C with increases of 327.4% and 99.9%, respectively (both *p* < 0.01), while blood glucose levels and the HOMA-IR index in the DE group were lower than in rats in group D with reductions of 40.6% and 15.6%, respectively (both *p* < 0.05), indicating that 8 weeks of aerobic exercise reduced insulin resistance and blood glucose levels in diabetic rats (Figure 1A).

We next monitored morphological changes in myocardial structure in HE-stained tissues using an optical microscope. Myocardial fibers in rats from group C were continuous and well organized, devoid of any signs of cell infiltration or vacuolar deformation, while myocardial cells from diabetic rats displayed bending deformations, infiltration of inflammatory cells, partial cell vacuolar degeneration, and accompanying microvascular congestion, as indicated by the arrows. After aerobic exercise, myocardial cell damage was restored in diabetic rats, with reduced cellular inflammation and myocardial microvascular congestion (Figure 1B), suggesting that 8 weeks of aerobic exercise improved the myocardial cell structure in diabetic rats.

### 3.2. Aerobic Exercise and Cardiac Function in Diabetic Rats

**Figure 2:**
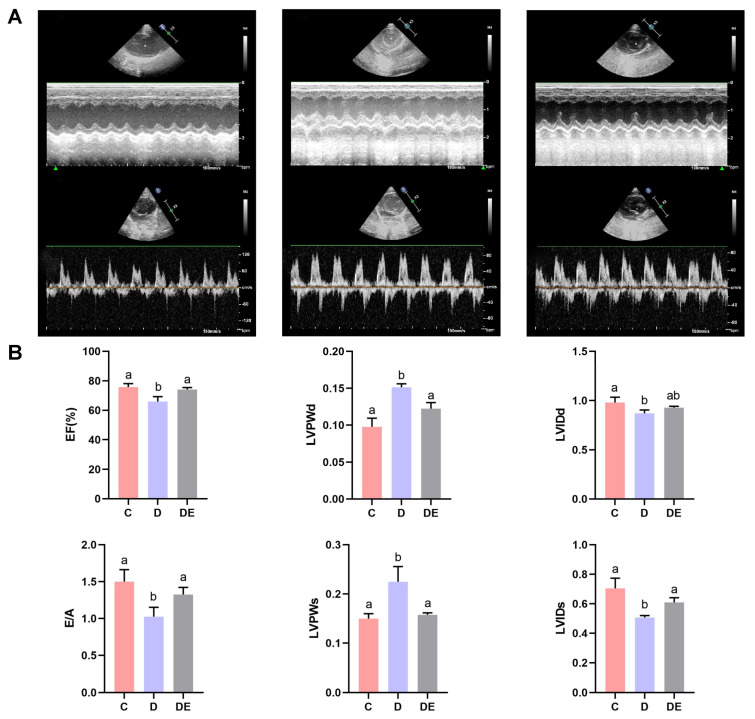
Effects of an 8-week aerobic exercise program on cardiac function in diabetic rats. (**A**) Representative M-mode echocardiographic images from rats in groups C, D, and DE. (**B**) Echocardiographic results indicated that diabetes diminished parameters of cardiac function such as EF, E/A, LVIDd; LVIDs (*p* < 0.05). Exercise increased all these parameters (*p* < 0.05), except for LVIDd, which remained unchanged (*p* > 0.05). LVPWd and LVPW values rats from group D were increased compared to those in group C (*p* < 0.05); exercise reversed these changes (*p* < 0.05). Different letters indicate significant differences between groups (*p* < 0.05), groups with the same letter are not significantly different (*p* > 0.05). EF = ejection fraction; E/A = early-to-late diastolic filling velocity ratio; LVIDd = left ventricular end-diastolic internal diameter; LVIDs = left ventricular end-systolic internal diameter; LVPWd = left ventricular posterior wall end-diastolic thickness; LVPWs = left ventricular posterior wall end-systolic thickness. Numerical values are presented as means ± standard deviation. C, control group; D, diabetic group; DE, diabetic exercise group.

We used M-mode echocardiography to evaluate multiple parameters of cardiac function in diabetic rats, as shown by representative echocardiographic images from all rat groups (Figure 2A). There were alterations in all parameters of cardiac systolic dysfunction, such as EF, LVIDd, LVIDs, LVPWd, and LVPWs, in rats from group D compared to cardiac tissue from group C (both *p* < 0.05). Exercise reversed these changes (both *p* < 0.05), except for LVIDd (*p* > 0.05) (Figure 2B). Diabetic rats had decreases in E/A ratios (a reflection of cardiac diastolic function), indicating impaired diastolic function (*p* < 0.01). Exercise improved the E/A ratios (*p* < 0.05). These findings collectively imply that aerobic exercise reduced cardiac dysfunction in rats with T2DM (Figure 2B).

### 3.3. Aerobic Exercise and Myocardial Metabolites in Diabetic Rats

#### 3.3.1. Multivariate Statistical Analysis

**Figure 3:**
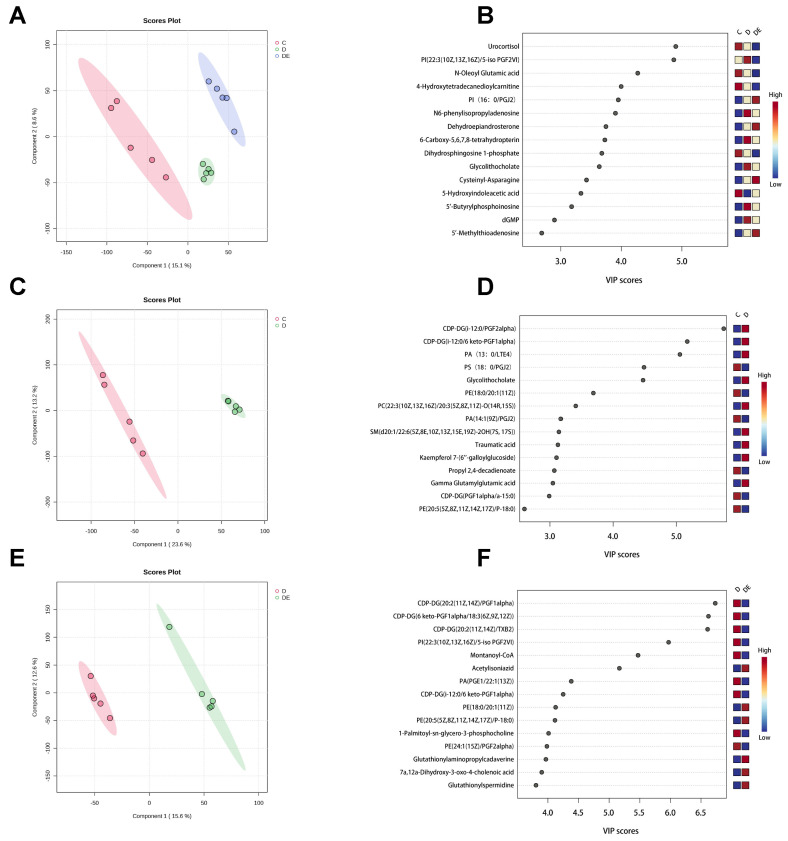
Multivariate statistical analysis of myocardial metabolomics in rats from the C, D, and DE groups. (**A**,**C**,**E**) PLS-DA score plots. There were clear separation trends among all groups, indicating significant differences in metabolites between different groups. (**B**,**D**,**F**) VIP score plots. VIP values indicate that phospholipid metabolites have a significant contribution in the comparisons between different groups. PLS-DA = partial least squares discriminant analysis; VIP = the variable importance in the projection. C, control group; D, diabetic group; DE, diabetic exercise group.

A non-targeted metabolomics approach was used to analyze the metabolic profiles of myocardial samples from rats in the study groups using both positive and negative ion modes. Multivariate statistical analysis was conducted using the principal component analysis (PCA) (Figure S1A) and partial least squares discriminant analysis (PLS-DA) (Figure 1A) supervised mode, disclosing distinct clustering in rats from groups C, D, and DE. Additionally, after 200 permutation tests, no over-fitting was detected in the PLS-DA model (Figure S1B). The variable importance in the projection (VIP) score plot revealed numerous substances in this model, greatly augmenting the metabolic distinctions among the groups. Considering the group separation, we ranked both components. Notably, substances with VIP values > 4 included urocortisol, phosphatidylinositols (22:3(10Z,13Z,16Z)/5-iso PGF2VI), and N-oleoyl glutamic acid (Figure 3B).

To gain a deeper understanding of alteration in myocardial metabolism in rats with diabetes and those exposed to aerobic exercise, we carried out separate PLS-DA analyses for the rats in groups C, D and D, DE. A distinct separation is visible when comparing groups C and D (Figure 3C), with no indication of over-fitting as confirmed by the permutation test (Figure S1C). Several substances associated with phospholipid metabolism, such as CDP-DG(i-12:0/PGF2-α), CDP-DG(i-12:0/6 keto-PGF1-α), and PA(13:0/LTE4), contributed substantially (VIP > 5) to this separation (Figure 3D). Similarly, under the PLS-DA supervised mode, the D and DE groups also showed a distinct separation (Figure 3E), again with no evidence of over-fitting according to the permutation test (Figure S1D). Metabolites with high contributions were also closely related to phospholipid metabolism, including CDP-DG(20:2(11Z,14Z)/PGF1-α), CDP-DG(6 keto-PGF1-α/18:3(6Z,9Z,12Z)), and CDP-DG(20:2(11Z,14Z)/TXB2) (VIP > 6) (Figure 3F).

#### 3.3.2. Volcano Diagram and Differential Metabolites

**Figure 4:**
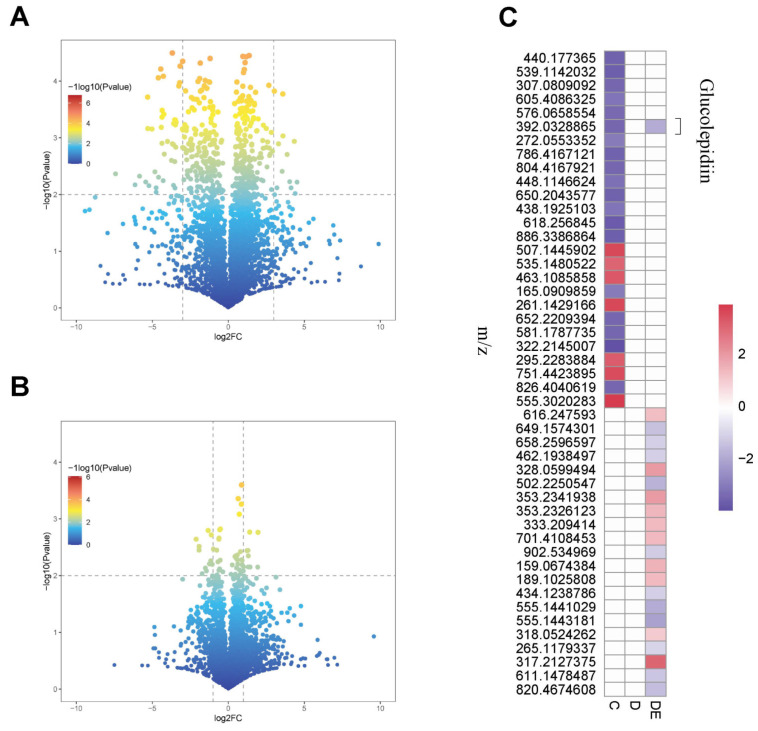
Differential metabolic in rats from groups D, C and D, DE. (**A**,**B**) Volcano plots displaying differential metabolites between D, C and D, DE groups, with 61 shared differential metabolites between the C and D groups and 22 shared differential metabolites between the D and DE groups. (**C**) Heatmap representing the average levels of metabolites, with only one differential metabolite, glucolepidiin, that changed in all three groups. C, control group; D, diabetic group; DE, diabetic exercise group.

We further assessed the impact of aerobic exercise on myocardial metabolic characteristics of diabetic rats using volcano plots. There were 12 metabolites that were upregulated and 49 metabolites that were downregulated in rats from group C compared to rats in group D (Figure 4A). Rats in group DE had 22 metabolites that were changed compared to rats in group D, with 10 metabolites upregulated and 12 metabolites downregulated (Figure 4B). We subsequently analyzed these intergroup differential metabolites, most of which changed independently, except for glucolepidiin (Figure 4C).

#### 3.3.3. Venn Diagram and Differential Metabolites

**Figure 5:**
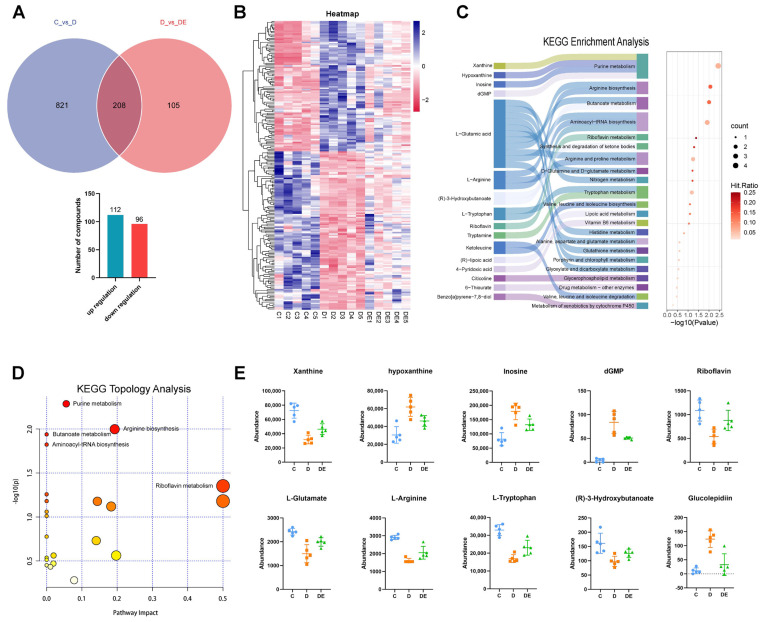
Effects of aerobic exercise on myocardial metabolism in diabetic rats. (**A**) Venn diagram showing the number of differential metabolites in groups C and D vs. D and DE. A total of 208 differential metabolites were identified, including 112 upregulated and 96 downregulated. (**B**) Cluster heatmap analysis of differential metabolites between groups. Metabolites in each group exhibited distinct clustering that can be separated. Blue indicates increased metabolite levels, and red signifies decreased levels. (**C**) KEGG enrichment analysis of differential metabolites. On the left, a Sankey diagram illustrates the connection between metabolites and metabolic pathways, and on the right, metabolites are arranged based on *p*-values from the enrichment analysis results. The primary enrichment is observed in metabolic pathways like purine metabolism and arginine biosynthesis. (**D**) Topological analysis of metabolites. A total of 23 metabolic pathways were identified, with 5 pathways having *p* < 0.05, including purine metabolism, arginine biosynthesis, butanoate metabolism, aminoacyl-tRNA biosynthesis, and riboflavin metabolism. (**E**) Selection of 10 potential metabolic markers through topological analysis and volcano plot analysis, along with their concentration changes listed. C, control group; D, diabetic group; DE, diabetic exercise group.

The Venn diagram illustrates the intersection of metabolites across the three groups (C_vs._D, D_vs._DE), revealing a total of 208 differential metabolites, of which 112 were upregulated and 96 were downregulated, indicating that 8 weeks of aerobic exercise altered 208 endogenous metabolites in diabetic rats (Figure 5A). The detailed information of the metabolites is provided in the Excel file (Table S1). Hierarchical clustering analysis indicated the changing patterns of differential metabolites by visually displaying alterations in metabolites related to exercise induced reductions in cardiac dysfunction. Hierarchical clustering analysis was performed on the 208 differential metabolites, where items marked in blue represented significantly increased metabolites, and those marked in red represented metabolites that were significantly decreased (Figure 5B). The heatmap of metabolites revealed distinct clustering, indicating the reliability of the selected metabolites (Figure 5B). We subsequently performed metabolite enrichment analysis and ranked them based on the magnitudes of *p*-values. Differential metabolites were enriched in pathways related to purine metabolism and arginine biosynthesis (Figure 5C).

Finally, we imported the 208 differential metabolites into MetaboAnalyst 5.0 for topological analysis; this showed that these metabolites were involved in 23 metabolic pathways. Using *p* < 0.05 as the threshold for filtering metabolic pathways, a total of five pathways affecting cardiac dysfunction in diabetic rats were identified: purine metabolism, arginine biosynthesis, butanoate metabolism, aminoacyl-tRNA biosynthesis, and riboflavin metabolism (Figure 5D). These five pathways collectively comprised nine metabolites that could have important roles in exercise-induced improvements of diabetic cardiac function (Figure 5E).

#### 3.3.4. Screening of Metabolic Markers

**Figure 6:**
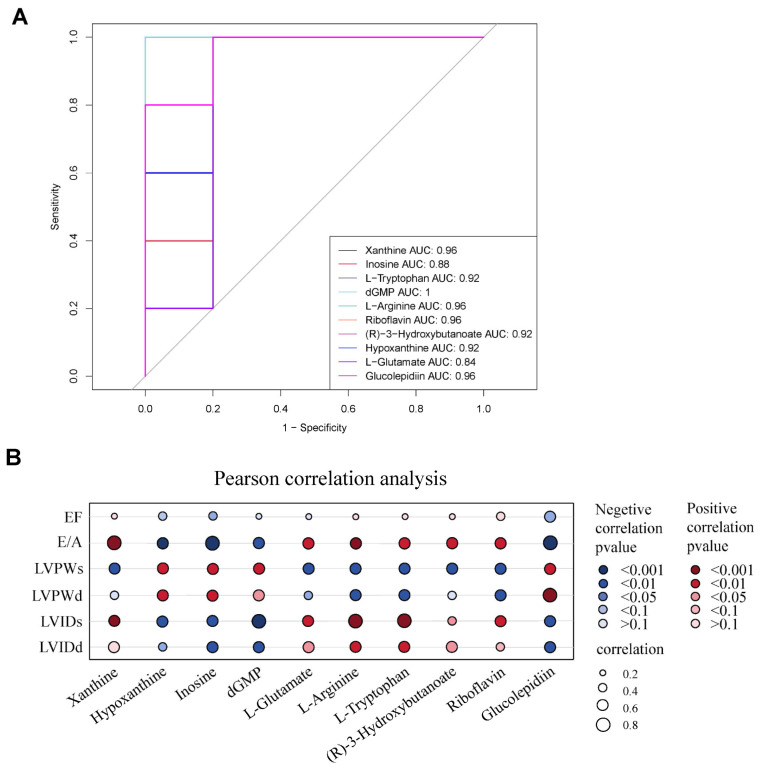
Validation of metabolic markers and their correlation with cardiac function. (**A**) ROC validation of metabolic markers, indicating that all AUC values are greater than 0.8. (**B**) Pearson correlation analysis was employed to ascertain the relationship between myocardial metabolic markers and heart function. Numerous metabolic markers demonstrated robust correlations with specific heart function indicators. Xanthine, inosine, and glucolepidiin exhibited strong correlations with the E/A. dGMP, arginine, and tryptophan demonstrated strong correlations with LVIDs, and glucolepidiin showed a strong correlation with LVPWd. The size of the circles in he figure denotes the strength of the correlation, where red signifies a positive correlation between metabolic markers and heart function indicators, while blue denotes a negative correlation. The intensity of color reflects the significance of the correlation. ROC = the receiver operating characteristic curve; AUC = ROC area under the curve; EF = ejection fraction; E/A = early-to-late diastolic filling velocity ratio; LVIDd = left ventricular end-diastolic internal diameter; LVIDs = left ventricular end-systolic internal diameter; LVPWd = left ventricular posterior wall end-diastolic thickness; LVPWs = left ventricular posterior wall end-systolic thickness.

ROC curves were used to evaluate the diagnostic value of metabolites for specific diseases, where ROC area under the curve (AUC) values evaluated the diagnostic value of myocardial metabolites in diabetic rats following aerobic exercise. ROC analysis was performed on 10 selected metabolites with AUC values greater than 0.8 [23]. The specific AUC values for these metabolites were xanthine (AUC = 0.96), hypoxanthine (AUC = 0.92), inosine (AUC = 0.88), dGMP (AUC = 1), L-glutamic acid (AUC = 0.84), L-arginine (AUC = 0.96), L-tryptophan (AUC = 0.92), (R)-3-hydroxybutyric acid (AUC = 0.92), riboflavin (AUC = 0.96), and glucolepidiin (AUC = 0.96). These findings indicate that these metabolites could have reliable robust diagnostic functions (Figure 6A).

#### 3.3.5. Correlation Analysis between Metabolic Markers and Cardiac Function

**Figure 7:**
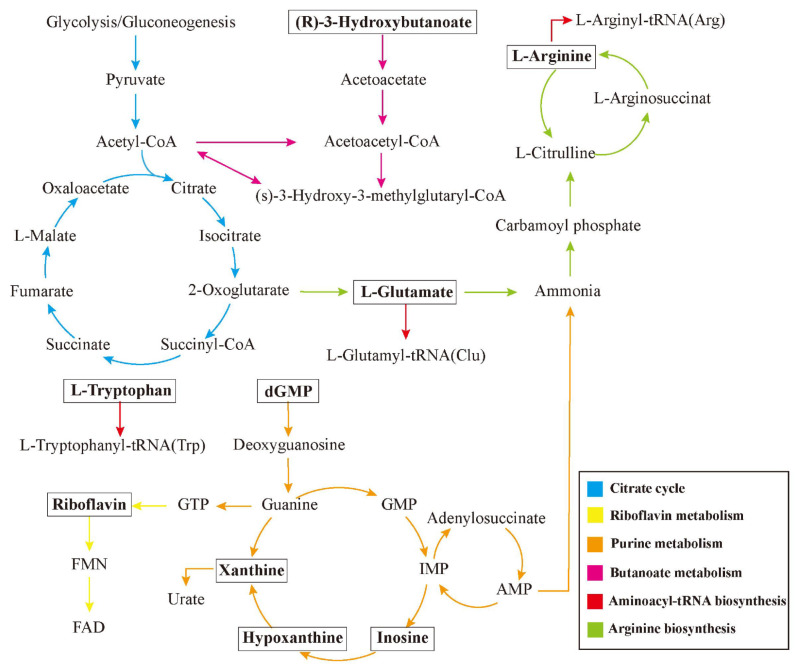
Metabolic pathway map. The selected metabolic markers (shown in bold in text boxes) were integrated based on the KEGG database to visualize their interconnections. The arrows in the diagram indicate the conversion direction from one metabolite to another, involving multiple metabolic pathways such as the citrate cycle, purine metabolism, and riboflavin metabolism. Different colors in the chart are used to distinguish each metabolic pathway. Overall, these metabolites are mostly involved in different parts of amino acid metabolism, energy metabolism, and purine metabolism, and they may be interconnected through these metabolic networks. This relationship in the conversion and function of metabolites is likely closely related to the improvement of cardiac function in diabetes by exercise.

We further investigated the relationship between differential metabolites and the improvement of cardiac function in diabetic hearts by performing a Pearson analysis of the relationship between the ten metabolic markers we identified and changes in cardiac functions. These results are shown in Figure 6, where larger circles indicate stronger correlations, red circles signify positive correlations and blue circles represent negative correlations. When examining correlations surpassing 0.8 [24], we identified a strong association between xanthine, inosine, glucolepidiin, and the cardiac E/A, indicating a substantial correlation between these three metabolites and cardiac diastolic function. Additionally, dGMP, L-arginine, and L-tryptophan were related to LVIDs, while glucolepidiin was related to LVPWd, implying a significant association between these four substances and cardiac systolic function (Figure 6B). Finally, we used metabolites to draw a metabolic pathway map (Figure 7).

## 4. Discussion

**Figure 8:**
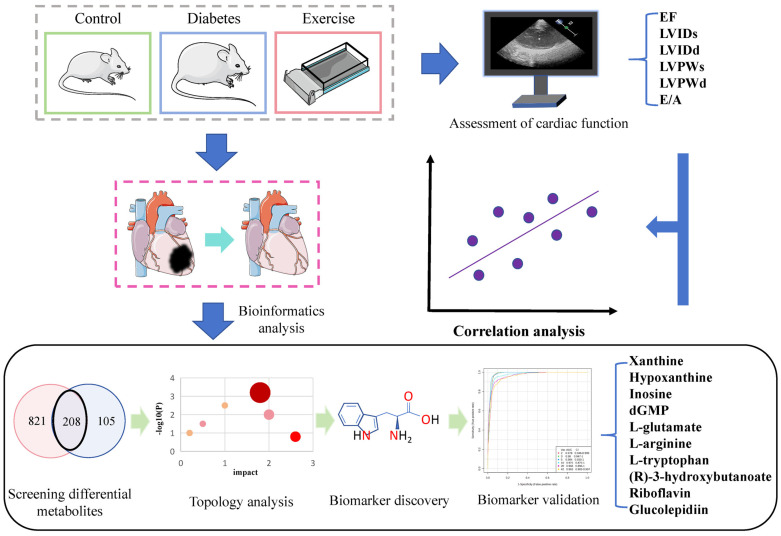
Metabolomics reveals that aerobic exercise alleviates cardiac dysfunction by altering myocardial metabolic markers in diabetic rats. Type 2 diabetic rats, successfully modeled using a high-fat, high-sugar diet, underwent echocardiographic assessment of cardiac function following 8 weeks of aerobic exercise. After the experience, myocardial tissue was extracted, and non-targeted metabolomics was used to analyze myocardial metabolites. A total of 208 differential metabolites were identified by comparing the myocardial metabolites across groups. Through topological analysis and receiver operating characteristic curve testing, 10 metabolites were selected. Finally, a Person correlation analysis revealed a relationship between the metabolites and cardiac function.

This study used a metabolomics approach to identify changes in diabetic cardiac dysfunction following long-term aerobic exercise, with a focus on metabolic pathways and biomarkers. We subsequently conducted a ROC validation correlation analysis of these biomarkers. The findings indicate that aerobic exercise modified five metabolic pathways with ten potential metabolic biomarkers in myocardial tissues of diabetic rats. A Pearson correlation analysis indicated a correlation between some biomarkers and cardiac function, providing a theoretical foundation for enhancing cardiac function in diabetic patients through aerobic exercise (Figure 8).

Purine metabolism describes the synthesis and degradation of purine nucleotides, as well as the regulation of guanosine and adenosine nucleotide pools, which are responsible for maintaining intracellular ATP and GTP concentrations [25]. Furthermore, GTP, acting as an intermediate in purine biosynthesis, participates in the initial stages of riboflavin (vitamin B2) synthesis [26]. Riboflavin, as a precursor of flavin mononucleotide (FMN) and flavin adenine dinucleotide (FAD), is an essential nutrient with roles in preventing myocardial injury and enhancing protein oxidative folding [26]. Previous studies indicated a link between riboflavin and the future risk of T2DM and its complications such as hypertension and coronary artery disease [27,28]. Heightened oxidative stress is associated with the development of diabetes, as shown by increased lipid peroxidation and protein carbonylation in STZ-induced diabetes rats [29]. Nevertheless, riboflavin treatment decreases lipid peroxidation and protein carbonylation, in addition to elevating levels of cardiac heme oxygenase-1 (HO-1) and superoxide dismutase (SOD) levels [29]. Furthermore, riboflavin supplementation also reduces the development of cardiac injury markers (CK and LDH) in diabetic rats, potentially preventing left ventricular hypertrophy and improving diastolic function [29]. Our study indicates that aerobic exercise restores decreased riboflavin levels in the myocardium of diabetic rats, with a positive relationship between riboflavin levels and diastolic function.

The monophosphate forms of purines transform into inosine during the catabolism of purines; inosine is then converted to hypoxanthine by purine nucleoside phosphorylase. Xanthine oxidase then converts hypoxanthine into xanthine, ultimately resulting in the production of uric acid [30]. It is widely recognized that purine metabolism is closely associated with the onset and progression of diabetes. Purine metabolite levels in human plasma are related to T2DM, with increased concentrations of inosine, hypoxanthine, and xanthine in the plasma and liver of diabetic patients [30]. Additionally, purine metabolites can act as markers for evaluating the severity of myocardial ischemia and myocardial infarction, as diabetic patients with acute myocardial infarction have increased plasma levels of inosine and hypoxanthine, but with no changed in xanthine levels [31]. Our study indicates that diabetic rats have increased levels of inosine and hypoxanthine in the myocardium, with a significant reduction in xanthine levels, likely due to decreased levels of xanthine oxidase in the early stages of diabetes. Nevertheless, 8 weeks of aerobic exercise enhanced the levels of myocardial purine metabolites. Correlation analysis demonstrated a strong association between the levels of xanthine and inosine, and the E/A ratio in the heart, suggesting that exercise ameliorates the pathological processes contributing to diastolic dysfunction in the diabetic myocardium.

Metabolomic studies indicate that some amino acids, such as glutamate, alanine, and branched-chain amino acids, could serve as early biomarkers for the onset of diabetes [32]. Glutamate, a crucial excitatory neurotransmitter in the central nervous system, also has a key role in controlling the function and activity of pancreatic islet endocrine cells, where it serves as a messenger for stimulating insulin release by pancreatic β-cells [33]. Abnormal glutamate levels cause harm to pancreatic islet cells and increase insulin resistance [34]. Persistent hyperglycemia results in vascular damage, leading to myocardial ischemia and cardiac dysfunction [35]. Nevertheless, during myocardial ischemia, glutamate assumes particular significance as it enhances the mechanical performance of the ischemic myocardium [36]. Additionally, glutamate is one of the key components in the synthesis of glutathione (GSH), which plays a crucial role in antioxidation. A deficiency in GSH can lead to oxidative stress, which is a fundamental mechanism in aging and many metabolic diseases, including T2DM [37]. Oxidative stress is also one of the primary causes of cardiac dysfunction in diabetic patients, resulting from both excessive production of reactive oxygen species (ROS) and impaired endogenous antioxidant mechanisms [38,39]. Numerous studies have shown that exercise training exerts antioxidant effects [40]. In our previous research, although aerobic exercise did not increase the activity of GSH in the myocardium of diabetic rats, it did reduce the production of myocardial ROS and enhance catalase activity, indicating that aerobic exercise plays an important role in combating oxidative stress [41].

Our analysis indicates that aerobic exercise increases arginine synthesis. An important role for arginine is as a substrate in the generation of nitric oxide (NO), a vasoactive substance released by the vascular endothelium. Tryptophan plays a role in regulating body weight and higher levels are linked to the risk of obesity and T2DM [42]. Tryptophan participates in three metabolic pathways within the body: the indole pathway, the kynurenine (Kyn) pathway, and the serotonin (5-HT) pathway [43]. Among these pathways, 5-HT can stimulate the growth of pancreatic β- cells and enhance insulin production [44]. In the Trp-Kyn pathway, the rate-limiting enzyme, indoleamine 2,3-dioxygenase 1 (IDO1), diminishes insulin resistance by reducing inflammation, suggesting that tryptophan metabolism reduces the complications of T2DM [45]. Furthermore, reduced tryptophan levels are inversely associated with cardiovascular diseases, including atherosclerosis and heart failure [46,47]. The 5-HT metabolic pathway is important in diabetes-related angiogenesis, as 5-HT influences blood flow velocity, and its receptors (5-HTR2A and 5-HTR1B) govern vascular dilation and constriction [48]. Our study indicates that aerobic exercise elevates the myocardial amino acids (glutamate, arginine, and tryptophan) of diabetic rats, and that these alterations are strongly associated with cardiac function. As a result, exercise enhances diabetic cardiac function by modifying the amino acid metabolic profile in diabetic cardiac tissue.

Hydroxybutyric acid (3HB) is a ketone body generated by the liver and is primarily used by energy-demanding tissues such as the central nervous system and the heart, where it is converted to acetyl-CoA for energy production [49]. In addition, 3HB also serves as a signaling molecule of cellular functions [50]. Initial data suggested that 3HB reduces blood glucose levels in patients with T2DM, and further investigations indicated a therapeutic effect of 3HB in T2DM through phosphorylation of peroxisome proliferator-activated receptor γ (PPARγ) at Ser273 in the adipose tissue of mice with T2DM, altering the expression of PPARγ regulatory factors to decrease insulin resistance [51]. Additionally, 3HB enhances cardiovascular function. Intravenous administration of 3HB improves cardiac output and ejection fraction in patients with chronic heart failure [52]. These reports agree with our findings that prolonged aerobic exercise mimics a fasting state (in terms of metabolism) by elevating ketone body concentrations and their utilization in the body [49]. Therefore, exercise-induced increases in 3HB levels can enhance cardiac function in T2DM.

Under normal conditions, the heart primarily relies on the oxidation of fatty acids and glucose for energy. In patients with diabetes, the levels of free fatty acids and triglycerides are significantly elevated, accompanied by an increase in fatty acid utilization by the myocardium. However, the myocardial fatty acid oxidation capacity is reduced, resulting in the accumulation of excessive fatty acids and their derivatives in the myocardium, which ultimately leads to structural and functional abnormalities in the heart [53,54]. Additionally, metabolomics data indicate that in the myocardium of STZ-induced rats, a significant increase in the types of long-chain acylcarnitines was observed, which is associated with impaired fatty acid β-oxidation. This suggests that diabetes leads to a reduced rate of myocardial fatty acid oxidation and the development of myocardial lipotoxicity [55]. Although numerous studies over the past 20 years have shown that exercise can promote lipid oxidation by enhancing the activity of lipases, it remains challenging to determine the exact changes in myocardial energy substrate utilization resulting from long-term exercise [56]. Long-term exercise training has been shown to increase the activity of hydroxyacyl-coenzyme A dehydrogenase (HADH) and carnitine palmitoyl transferase 1 (CPT1) in mouse myocardium, along with enhanced expression of peroxisome proliferator-activated receptor α (PPARα) and clusters of differentiation (CD36) genes [57]. However, other studies have found that exercise does not alter factors associated with myocardial fatty acid oxidation, such as CPT1b, PPARα, and CD36 [58,59]. Similarly, our research did not identify any significant effects of long-term aerobic exercise on pathways related to myocardial lipid metabolism. Future studies might consider employing different exercise modalities or intensities to further evaluate the impact of exercise therapy on myocardial fatty acid oxidation in diabetic conditions.

It is important to recognize that non-targeted metabolomics, which has many advantages, also has some drawbacks. As an unbiased detection method, it can broadly identify various metabolites and their pathways, and is an effective and reliable method to discover disease biomarkers. However, as a tool, non-targeted metabolomics can only perform semi-quantitative analysis, which results in the detection of metabolites at relative concentrations rather than absolute concentrations. Such results can only reflect the relative levels of metabolites between different groups. Furthermore, although differential metabolites provide useful information, the lack of quantitative data may limit the understanding of the biological processes involved in metabolic pathways. In mass spectrometry analysis, in-source fragmentation is a common phenomenon that can lead to the formation of ion fragments during the ionization of target metabolites, potentially complicating their identification and resulting in the overinterpretation of metabolic data. There, future research should involve targeted metabolomic analysis of the biomarkers we have identified to obtain more accurate data. Additionally, one of the limitations of this study is that the PLS-DA model explains only a small percentage of the overall data variance. This limited explanatory power might affect the accuracy and generalizability of the model’s predictions. Future studies should consider using more complex or appropriate models to enhance the explanatory power of the results. Secondly, the relatively small sample size in our experiments may limit detailed exploration of cardiac function in diabetes, and future studies with larger sample sizes will validate our findings. Finally, correlation analysis is a commonly used and highly efficient tool in metabolomics, enabling data mining and the rapid identification of meaningful metabolites. By correlating metabolites with specific traits, researchers can gain insights into the biological processes associated with particular metabolites, thus providing direction for subsequent studies and further exploration. However, it is important to acknowledge the potential limitations of using correlation analysis in the context of metabolomics. Firstly, while correlation analysis can reveal associations between metabolites and traits, it does not exclude the involvement of other metabolites in these processes. Secondly, due to the limited sample size in this study, the analysis may not capture associations between low-abundance but biologically significant metabolites and specific traits. Additionally, in metabolomics, a particular metabolite may not only be associated with a single trait but could also be involved in other biological processes. Therefore, over-reliance on correlation analysis might obscure the broader biological functions of metabolites, potentially overlooking their roles in other processes. These limitations could impact the interpretation of the results, and as such, it is crucial to recognize these constraints and approach conclusions with caution.

## 5. Conclusions

Our study demonstrates that continuous aerobic exercise for 8 weeks changes myocardial metabolites in diabetic rats and improves cardiac dysfunction by altering metabolic pathways such as purine metabolism and arginine biosynthesis. Furthermore, metabolites including xanthine, hypoxanthine, inosine, dGMP, L-glutamic acid, L-arginine, L-tryptophan, (R)-3-hydroxybutyric acid, riboflavin, and glucolepidiin could serve as key representative biomarkers for the improvement of T2DM-related cardiac dysfunction by aerobic exercise. Our findings provide some potential mechanisms underlying cardiac damage and indicate that aerobic exercise can prevent cardiac injury in T2DM.

## Data Availability

Data are contained within the article and Supplementary Materials.

## Supplementary Materials

The following supporting information can be downloaded at https://www.mdpi.com/article/10.3390/antiox13101167/s1, Table S1: Raw metabolomics data; Figure S1: PCA plot and PLS-DA permutation test plot.

## References

[1] Donath M.Y., Shoelson S.E. (2011). Type 2 diabetes as an inflammatory disease. Nat. Rev. Immunol..

[2] Sampath Kumar A., Maiya A.G., Shastry B.A., Vaishali K., Ravishankar N., Hazari A., Gundmi S., Jadhav R. (2019). Exercise and insulin resistance in type 2 diabetes mellitus: A systematic review and meta-analysis. Ann. Phys. Rehabil. Med..

[3] Karwi Q.G., Ho K.L., Pherwani S., Ketema E.B., Sun Q., Lopaschuk G.D. (2022). Concurrent diabetes and heart failure: Interplay and novel therapeutic approaches. Cardiovasc. Res..

[4] Ho K.L., Karwi Q.G., Connolly D., Pherwani S., Ketema E.B., Ussher J.R., Lopaschuk G.D. (2022). Metabolic, structural and biochemical changes in diabetes and the development of heart failure. Diabetologia.

[5] Jankauskas S.S., Kansakar U., Varzideh F., Wilson S., Mone P., Lombardi A., Gambardella J., Santulli G. (2021). Heart failure in diabetes. Metabolism.

[6] Jin L., Geng L., Ying L., Shu L., Ye K., Yang R., Liu Y., Wang Y., Cai Y., Jiang X. (2022). FGF21-Sirtuin 3 Axis Confers the Protective Effects of Exercise Against Diabetic Cardiomyopathy by Governing Mitochondrial Integrity. Circulation.

[7] Kirwan J.P., Sacks J., Nieuwoudt S. (2017). The essential role of exercise in the management of type 2 diabetes. Cleve. Clin. J. Med..

[8] Mann N., Rosenzweig A. (2012). Can exercise teach us how to treat heart disease?. Circulation.

[9] Moe B., Eilertsen E., Nilsen T.I. (2013). The combined effect of leisure-time physical activity and diabetes on cardiovascular mortality: The Nord-Trondelag Health (HUNT) cohort study, Norway. Diabetes Care.

[10] Seo D.Y., Ko J.R., Jang J.E., Kim T.N., Youm J.B., Kwak H.B., Bae J.H., Kim A.H., Ko K.S., Rhee B.D. (2019). Exercise as A Potential Therapeutic Target for Diabetic Cardiomyopathy: Insight into the Underlying Mechanisms. Int. J. Mol. Sci..

[11] Bidasee K.R., Zheng H., Shao C.H., Parbhu S.K., Rozanski G.J., Patel K.P. (2008). Exercise training initiated after the onset of diabetes preserves myocardial function: Effects on expression of beta-adrenoceptors. J. Appl. Physiol..

[12] Long J., Yang Z., Wang L., Han Y., Peng C., Yan C., Yan D. (2020). Metabolite biomarkers of type 2 diabetes mellitus and pre-diabetes: A systematic review and meta-analysis. BMC Endocr. Disord..

[13] Zhang F., Chen X., Yang M., Shen X., Wang Y., Zhong D., Zeng F., Jin R. (2024). Metabolic impairments associated with type 2 diabetes mellitus and the potential effects of exercise therapy: An exploratory randomized trial based on untargeted metabolomics. PLoS ONE.

[14] Jing L., Chengji W. (2019). GC/MS-based metabolomics strategy to analyze the effect of exercise intervention in diabetic rats. Endocr. Connect..

[15] Arneth B., Arneth R., Shams M. (2019). Metabolomics of Type 1 and Type 2 Diabetes. Int. J. Mol. Sci..

[16] Zhou Y., Men L., Pi Z., Wei M., Song F., Zhao C., Liu Z. (2018). Fecal Metabolomics of Type 2 Diabetic Rats and Treatment with Gardenia jasminoides Ellis Based on Mass Spectrometry Technique. J. Agric. Food Chem..

[17] Stalin A., Irudayaraj S.S., Gandhi G.R., Balakrishna K., Ignacimuthu S., Al-Dhabi N.A. (2016). Hypoglycemic activity of 6-bromoembelin and vilangin in high-fat diet fed-streptozotocin-induced type 2 diabetic rats and molecular docking studies. Life Sci..

[18] Gu H., Xia X., Chen Z., Liang H., Yan J., Xu F., Weng J. (2014). Insulin therapy improves islet functions by restoring pancreatic vasculature in high-fat diet-fed streptozotocin-diabetic rats. J. Diabetes.

[19] Al-Awar A., Kupai K., Veszelka M., Szűcs G., Attieh Z., Murlasits Z., Török S., Pósa A., Varga C. (2016). Experimental Diabetes Mellitus in Different Animal Models. J. Diabetes Res..

[20] Epp R.A., Susser S.E., Morissette M.P., Kehler D.S., Jassal D.S., Duhamel T.A. (2013). Exercise training prevents the development of cardiac dysfunction in the low-dose streptozotocin diabetic rats fed a high-fat diet. Can. J. Physiol. Pharmacol..

[21] Bedford T.G., Tipton C.M., Wilson N.C., Oppliger R.A., Gisolfi C.V. (1979). Maximum oxygen consumption of rats and its changes with various experimental procedures. J. Appl. Physiol. Respir. Environ. Exerc. Physiol..

[22] Matthews D.R., Hosker J.P., Rudenski A.S., Naylor B.A., Treacher D.F., Turner R.C. (1985). Homeostasis model assessment: Insulin resistance and beta-cell function from fasting plasma glucose and insulin concentrations in man. Diabetologia.

[23] Bhuiyan M.U., Blyth C.C., West R., Lang J., Rahman T., Granland C., de Gier C., Borland M.L., Thornton R.B., Kirkham L.S. (2019). Combination of clinical symptoms and blood biomarkers can improve discrimination between bacterial or viral community-acquired pneumonia in children. BMC Pulm. Med..

[24] van der Laan L., Cardenas A., Vermeulen R., Fadadu R.P., Hubbard A.E., Phillips R.V., Zhang L., Breeze C., Hu W., Wen C. (2022). Epigenetic aging biomarkers and occupational exposure to benzene, trichloroethylene and formaldehyde. Environ. Int..

[25] Dudzinska W., Lubkowska A., Dolegowska B., Safranow K., Jakubowska K. (2010). Adenine, guanine and pyridine nucleotides in blood during physical exercise and restitution in healthy subjects. Eur. J. Appl. Physiol..

[26] Iwanaga K., Hasegawa T., Hultquist D.E., Harada H., Yoshikawa Y., Yanamadala S., Liao H., Visovatti S.H., Pinsky D.J. (2007). Riboflavin-mediated reduction of oxidant injury, rejection, and vasculopathy after cardiac allotransplantation. Transplantation.

[27] Liu J., Wang L., Qian Y., Shen Q., Yang M., Dong Y., Chen H., Yang Z., Liu Y., Cui X. (2022). Metabolic and Genetic Markers Improve Prediction of Incident Type 2 Diabetes: A Nested Case-Control Study in Chinese. J. Clin. Endocrinol. Metab..

[28] Thakur K., Tomar S.K., Singh A.K., Mandal S., Arora S. (2017). Riboflavin and health: A review of recent human research. Crit. Rev. Food Sci. Nutr..

[29] Wang G., Li W., Lu X., Zhao X. (2011). Riboflavin alleviates cardiac failure in Type I diabetic cardiomyopathy. Heart Int..

[30] Varadaiah Y.G.C., Sivanesan S., Nayak S.B., Thirumalarao K.R. (2022). Purine metabolites can indicate diabetes progression. Arch. Physiol. Biochem..

[31] Al-Shamiri S.A., Hasan N.A., Frankul W.M., Al-Hamdi A.T. (2009). Purines and oxypurines in myocardial ischemia. Saudi Med. J..

[32] Wang S., Li M., Lin H., Wang G., Xu Y., Zhao X., Hu C., Zhang Y., Zheng R., Hu R. (2022). Amino acids, microbiota-related metabolites, and the risk of incident diabetes among normoglycemic Chinese adults: Findings from the 4C study. Cell Rep. Med..

[33] Otter S., Lammert E. (2016). Exciting Times for Pancreatic Islets: Glutamate Signaling in Endocrine Cells. Trends Endocrinol. Metab..

[34] Davalli A.M., Perego C., Folli F.B. (2012). The potential role of glutamate in the current diabetes epidemic. Acta Diabetol..

[35] Park J.J. (2021). Epidemiology, Pathophysiology, Diagnosis and Treatment of Heart Failure in Diabetes. Diabetes Metab. J..

[36] Du J., Li X.H., Li Y.J. (2016). Glutamate in peripheral organs: Biology and pharmacology. Eur. J. Pharmacol..

[37] Wu G., Fang Y.Z., Yang S., Lupton J.R., Turner N.D. (2004). Glutathione metabolism and its implications for health. J. Nutr..

[38] Jia G., Whaley-Connell A., Sowers J.R. (2018). Diabetic cardiomyopathy: A hyperglycaemia- and insulin-resistance-induced heart disease. Diabetologia.

[39] Nishikawa T., Edelstein D., Du X.L., Yamagishi S., Matsumura T., Kaneda Y., Yorek M.A., Beebe D., Oates P.J., Hammes H.P. (2000). Normalizing mitochondrial superoxide production blocks three pathways of hyperglycaemic damage. Nature.

[40] de Sousa C.V., Sales M.M., Rosa T.S., Lewis J.E., de Andrade R.V., Simões H.G. (2017). The Antioxidant Effect of Exercise: A Systematic Review and Meta-Analysis. Sports Med..

[41] Tang M., Su Q., Duan Y., Fu Y., Liang M., Pan Y., Yuan J., Wang M., Pang X., Ma J. (2023). The role of MOTS-c-mediated antioxidant defense in aerobic exercise alleviating diabetic myocardial injury. Sci. Rep..

[42] Wang W., Wang X., Liu L., Liu Z., Han T., Sun C., Yang X. (2022). Dietary tryptophan and the risk of obesity and type 2 diabetes: Total effect and mediation effect of sleep duration. Obesity.

[43] Legan T.B., Lavoie B., Mawe G.M. (2022). Direct and indirect mechanisms by which the gut microbiota influence host serotonin systems. Neurogastroenterol. Motil..

[44] Makhmutova M., Weitz J., Tamayo A., Pereira E., Boulina M., Almaça J., Rodriguez-Diaz R., Caicedo A. (2021). Pancreatic β-Cells Communicate with Vagal Sensory Neurons. Gastroenterology.

[45] Gao J., Yang T., Song B., Ma X., Ma Y., Lin X., Wang H. (2023). Abnormal tryptophan catabolism in diabetes mellitus and its complications: Opportunities and challenges. Biomed. Pharmacother..

[46] Sudar-Milovanovic E., Gluvic Z., Obradovic M., Zaric B., Isenovic E.R. (2022). Tryptophan Metabolism in Atherosclerosis and Diabetes. Curr. Med. Chem..

[47] Razquin C., Ruiz-Canela M., Toledo E., Hernández-Alonso P., Clish C.B., Guasch-Ferré M., Li J., Wittenbecher C., Dennis C., Alonso-Gómez A. (2021). Metabolomics of the tryptophan-kynurenine degradation pathway and risk of atrial fibrillation and heart failure: Potential modification effect of Mediterranean diet. Am. J. Clin. Nutr..

[48] Imamdin A., van der Vorst E.P.C. (2023). Exploring the Role of Serotonin as an Immune Modulatory Component in Cardiovascular Diseases. Int. J. Mol. Sci..

[49] Møller N. (2020). Ketone Body, 3-Hydroxybutyrate: Minor Metabolite—Major Medical Manifestations. J. Clin. Endocrinol. Metab..

[50] Newman J.C., Verdin E. (2017). β-Hydroxybutyrate: A Signaling Metabolite. Annu. Rev. Nutr..

[51] Zhang Y., Li Z., Liu X., Chen X., Zhang S., Chen Y., Chen J., Chen J., Wu F., Chen G.Q. (2023). 3-Hydroxybutyrate ameliorates insulin resistance by inhibiting PPARγ Ser273 phosphorylation in type 2 diabetic mice. Signal Transduct. Target.Ther..

[52] Nielsen R., Møller N., Gormsen L.C., Tolbod L.P., Hansson N.H., Sorensen J., Harms H.J., Frøkiær J., Eiskjaer H., Jespersen N.R. (2019). Cardiovascular Effects of Treatment With the Ketone Body 3-Hydroxybutyrate in Chronic Heart Failure Patients. Circulation.

[53] Buchanan J., Mazumder P.K., Hu P., Chakrabarti G., Roberts M.W., Yun U.J., Cooksey R.C., Litwin S.E., Abel E.D. (2005). Reduced cardiac efficiency and altered substrate metabolism precedes the onset of hyperglycemia and contractile dysfunction in two mouse models of insulin resistance and obesity. Endocrinology.

[54] Fukushima A., Lopaschuk G.D. (2016). Cardiac fatty acid oxidation in heart failure associated with obesity and diabetes. Biochim. Biophys. Acta.

[55] Schooneman M.G., Vaz F.M., Houten S.M., Soeters M.R. (2013). Acylcarnitines: Reflecting or inflicting insulin resistance?. Diabetes.

[56] Kolwicz S.C. (2018). An “Exercise” in Cardiac Metabolism. Front. Cardiovasc. Med..

[57] Riehle C., Wende A.R., Zhu Y., Oliveira K.J., Pereira R.O., Jaishy B.P., Bevins J., Valdez S., Noh J., Kim B.J. (2014). Insulin receptor substrates are essential for the bioenergetic and hypertrophic response of the heart to exercise training. Mol. Cell. Biol..

[58] Iemitsu M., Miyauchi T., Maeda S., Sakai S., Fujii N., Miyazaki H., Kakinuma Y., Matsuda M., Yamaguchi I. (2003). Cardiac hypertrophy by hypertension and exercise training exhibits different gene expression of enzymes in energy metabolism. Hypertens Res..

[59] Monleon D., Garcia-Valles R., Morales J.M., Brioche T., Olaso-Gonzalez G., Lopez-Grueso R., Gomez-Cabrera M.C., Viña J. (2014). Metabolomic analysis of long-term spontaneous exercise in mice suggests increased lipolysis and altered glucose metabolism when animals are at rest. J. Appl. Physiol..

