# Supplementary material for: Metabolomic Profiling Reveals That Exercise Lowers Biomarkers of Cardiac Dysfunction in Rats with Type 2 Diabetes
Source: Antioxidants (Basel). 2024 Sep 26;13(10):1167. doi: 10.3390/antiox13101167 (PMC11505272; doi:10.3390/antiox13101167)
Supplement: Supplementary file 1 [file antioxidants-13-01167-s001.zip › Figure S1. PCA plot and PLS-DA permutation test plot.pdf]

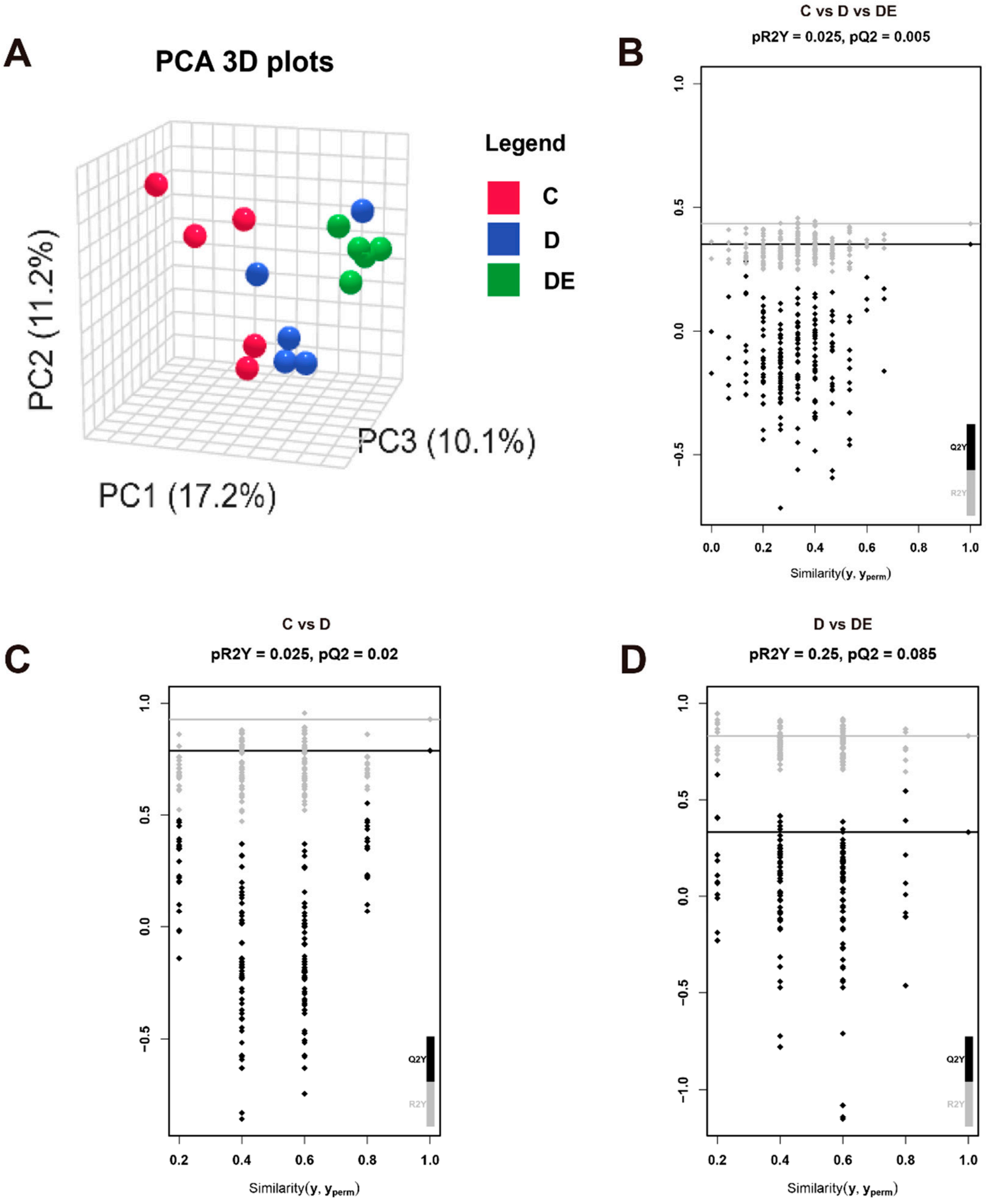

Figure S1. 3D plots of principal component analysis (PCA) and permutation test plots of Partial Least Squares Discriminant Analysis (PLS-DA) for different groups. (**A**) PCA 3D plot showing the separation between groups and the clustering within each group. (**B**–**D**) Permutation test plots after PLS-DA analysis. A total of 200 permutations were conducted after PLS-DA analysis, and the plots indicate that there is no overfitting of the data (pQ2 < 0.1). PCA = principal component analysis, PLS-DA = partial least squares discriminant analysis. C, control group; D, diabetic group; DE, diabetic exercise group.
